# Unilateral Nephrectomy Stimulates ERK and Is Associated With Enhanced Na Transport

**DOI:** 10.3389/fphys.2021.583453

**Published:** 2021-02-03

**Authors:** Robert Repetti, Nomrota Majumder, Karin Carneiro De Oliveira, Jennifer Meth, Tenzin Yangchen, Mukut Sharma, Tarak Srivastava, Rajeev Rohatgi

**Affiliations:** ^1^Northport VA Medical Center, Northport, NY, United States; ^2^School of Medicine, Stony Brook University, Stony Brook, NY, United States; ^3^Program in Public Health, School of Medicine, Stony Brook University, Stony Brook, NY, United States; ^4^Children’s Mercy Hospital, Kansas City, MO, United States; ^5^Kansas City VA Medical Center, Kansas City, MO, United States

**Keywords:** nephrectomy, sodium transport, mitogen activated protein kinase, cortical collecting duct, electrophysiology

## Abstract

Nephron loss initiates compensatory hemodynamic and cellular effects on the remaining nephrons. Increases in single nephron glomerular filtration rate and tubular flow rate exert higher fluid shear stress (FSS) on tubules. In principal cell (PC) culture models FSS induces ERK, and ERK is implicated in the regulation of transepithelial sodium (Na) transport, as well as, proliferation. Thus, we hypothesize that high tubular flow and FSS mediate ERK activation in the cortical collecting duct (CCD) of solitary kidney which regulates amiloride sensitive Na transport and affects CCD cell number. Immunoblotting of whole kidney protein lysate was performed to determine phospho-ERK (pERK) expression. Next, sham and unilateral nephrectomized mice were stained with anti-pERK antibodies, and dolichos biflorus agglutinin (DBA) to identify PCs with pERK. Murine PCs (mpkCCD) were grown on semi-permeable supports under static, FSS, and FSS with U0126 (a MEK1/2 inhibitor) conditions to measure the effects of FSS and ERK inhibition on amiloride sensitive Na short circuit current (*Isc*). pERK abundance was greater in kidney lysate of unilateral vs. sham nephrectomies. The total number of cells in CCD and pERK positive PCs increased in nephrectomized mice (9.3 ± 0.4 vs. 6.1 ± 0.2 and 5.1 ± 0.5 vs. 3.6 ± 0.3 cell per CCD nephrectomy vs. sham, respectively, *n* > 6 per group, *p* < 0.05). However, Ki67, a marker of proliferation, did not differ by immunoblot or immunohistochemistry in nephrectomy samples at 1 month compared to sham. Next, amiloride sensitive *Isc* in static mpkCCD cells was 25.3 ± 1.7 μA/cm^2^ (*n* = 21), but after exposure to 24 h of FSS the *Isc* increased to 41.4 ± 2.8 μA/cm^2^ (*n* = 22; *p* < 0.01) and returned to 19.1 ± 2.1 μA/cm^2^ (*n* = 18, *p* < 0.01) upon treatment with U0126. Though FSS did not alter α- or γ-ENaC expression in mpkCCD cells, γ-ENaC was reduced in U0126 treated cells. In conclusion, pERK increases in whole kidney and, specifically, CCD cells after nephrectomy, but pERK was not associated with active proliferation at 1-month post-nephrectomy. *In vitro* studies suggest high tubular flow induces ERK dependent ENaC Na absorption and may play a critical role in Na balance post-nephrectomy.

## Introduction

Nephron loss is accompanied by compensatory changes in the remaining nephrons to maintain (1) glomerular filtration rate (GFR), (2) ion and water balance, and (3) acid-base homeostasis. A “non-pathologic” model of nephron loss is unilateral nephrectomy which is accompanied by compensatory changes in the solitary kidney. Human studies show an increase in single kidney blood flow rate of ∼42% and single kidney GFR of ∼40% after a median of follow up time of 6.3 years; however, all of these changes occur during the first year post-nephrectomy (Lenihan et al., 2015). The rise in single kidney GFR is principally to due to proportionate increases in the whole kidney ultrafiltration coefficient (K_f_) which reflects the filtration surface area (Lenihan et al., 2015). Soon after nephrectomy the single kidney fractional excretion of Na (FNa) and the fractional excretion of potassium (FEK) nearly double to maintain homeostasis (Hayslett, 1979). The literature suggests most of these adaptive fluctuations in cation transport occur at the level of the distal tubule and collecting duct, but the biological mechanisms by which these changes are induced are not clear.

These adaptive physiologic changes in GFR, Na and K excretion are attended by cellular changes, including glomerular and tubular hypertrophy, as well as, by alterations in biomechanical forces, including augmented glomerular hydrostatic pressure and fluid shear stress (FSS) of podocytes and tubular epithelia (Srivastava et al., 2014). Biomechanical forces play an important regulatory role in the physiologic functions of the kidney. Glomerular hydrostatic and oncotic pressures are critical to form ultrafiltrate. The ultrafiltrate travels through the tubules and it exerts pressure, leading to circumferential stretch (CS) and FSS along the apical plasma membrane. In cases where single nephron GFR rises, as in unilateral nephrectomy, these biomechanical forces will increase.

Biomechanical forces play an important role to stimulate the compensatory physiologic changes in the solitary kidney after nephrectomy. Rising tubular flow and FSS in proximal tubule (PT), thick ascending limb (TAL), and cortical collecting duct (CCD) augment cation transport in their respective segments, presumably to protect against Na losses which is a greater short-term risk for the organism’s survival than Na retention (Muto et al., 1994; Du et al., 2004, 2006; Morimoto et al., 2006; Hong and Garvin, 2014). Though flow-mediated Na absorption prevents renal Na losses, flow mediated paracrine factors are stimulated to inhibit Na absorption and enhance Na excretion. These factors include nitric oxide (NO), endothelin-1 (ET1), nucleotides, prostanoids, and heme oxygenase-1 (HO1) (Rieg et al., 2007; Lyon-Roberts et al., 2011; Flores et al., 2012; Liu et al., 2015; Pandit et al., 2015; Gao et al., 2018; Repetti et al., 2019). Therefore, net Na excretion from each segment represents a balance of flow-mediated Na absorption vs. flow-mediated paracrine molecules that antagonize Na transport (Flores et al., 2012).

Though glomerular and tubular hypertrophy are classic renal responses to nephrectomy, cellular proliferation also occurs as illustrated by increased DNA content post-nephrectomy in 5 week old rodents (Terzi et al., 1995). Other investigators have shown in young rodents that the number cells per kidney is increased at 3 weeks and 3 months post-nephrectomy compared to sham control, implying a proliferative response; however, the affected tubular segments are unknown (Soukupova et al., 1975). Because the distal nephron and, in particular, the CCD play critical roles in Na and K excretion post-nephrectomy, structural and physiologic changes may occur. Moreover, FSS, as least *in vitro*, induces mitogen activated protein kinase (MAPK) activity, specifically ERK and p38, in mpkCCD cells, a mouse principal cell (PC) line (Carrisoza-Gaytan et al., 2014). Of interest, CS had no effect on ERK phosphorylation and induced a modest reduction in p-38 phosphorylation (Carrisoza-Gaytan et al., 2014). This led us to hypothesize that nephrectomy in the mouse may lead FSS mediated ERK stimulation in the CCD which can be a regulator of transepithelial cation transport and proliferation.

## Materials and Methods

### Animals

Studies of mice were carried out with approval of the Institutional Animal Care and Use Committee (IACUC), Safety Subcommittee, and R&D Committee as the Veterans Affairs Medical Center (Kansas City, MO, United States). Male sv129 mice (13–14 week) from Charles Rivers (Indianapolis, IN, United States) were purchased and maintained at the Association for Assessment and Accreditation of Laboratory Care-approved facilities with unrestricted access to food and water under 12:12-h light-dark cycles.

### Unilateral Nephrectomy

13 to 14-week-old mice underwent surgical removal of the right kidney. Another set of 13 to 14-week-old sv129 mice underwent a sham operation for experimental control. Four weeks later, the left kidney was harvested from all animals for analysis. Kidneys were fixed in 10% formalin, embedded in paraffin and sectioned at 3–5 μm for immunohistology and immunofluorescence staining.

### Immunohistology and Immunofluorescence

For fluorescence studies, the Zeiss upright epifluorescence microscope with 10× and 40× objectives visualized kidney sections and the AxioVision 4.8 software imaged these sections. The AxioVision 4.8 enabled measurements of tubular diameter in microns. For immunohistochemical renal imaging a Nikon Eclipse Ci upright microscope with 10× eyepiece and 20× or 40× objectives was used for visualization and the Nikon Elements imaging software used for imaging.

Citrate based antigen retrieval was performed on kidney sections prior to immunolabelling. A goat anti-rabbit antibody conjugated to Alexa 488 was used to localize the rabbit polyclonal anti-phospho-ERK (pERK) antibody and a dolichos biflorus agglutinin (DBA) conjugated to rhodamine was used to identify PCs of the CCD. Ten CCDs were identified in each kidney, sham (*n* = 6) or nephrectomy (*n* = 7). The short diameter of each CCD was measured. The number of cells, of DBA stained cells, of pERK stained cells, and of dual DBA and pERK costained cells were counted in each CCD. The average of each parameter was calculated for sham and nephrectomy kidney, and each kidney is reflected as an independent experiment.

### Cell Culture

Murine immortalized mpkCCD cells were grown in DMEM:Ham’s F12 (with 60 nM sodium selenate, 5 μg/ml transferrin, 2 mM glutamine, 50 nM dexamethasone, 1 nM triiodothyronine, 10 ng/ml epidermal growth factor, 5 μg/ml insulin, 20 mM D-glucose, 2% fetal calf serum, and 20 mM HEPES) on collagen coated, polycarbonate snapwell inserts with a pore size of 0.4 μ (Corning) or grown six well plastic plates. Cells were grown to confluence in 5–6 days and then the media replaced by media containing charcoal stripped serum for 24 h. Next, the mpkCCD cells are made serum free with 10 μM aldosterone for 24 h. MpkCCD cells exposed to FSS were placed on a rotator at 27 revolutions/min to generate FSS of 0.4 dynes/cm^2^, according to FFS (dynes/cm^2^) = $a\,\sqrt{n\,\rho \,{\left(2\,\pi \,f\right)}^{3}}$ where *a* is the radius of the orbital shaker (*n* = 16 mm), *n* is the density of the media, ρ is the viscosity of media, and *f* is the frequency of rotations (rpm) (Ernandez et al., 2018). Cells were used only up to passage 15 due to the risk of genetic drift.

### Ussing Chamber Electrophysiology

Cell monolayers were mounted between the Lucite half chambers of the Ussing chamber (Physiological Instruments, San Diego, CA, United States) for electrophysiological studies. Cell monolayers were bathed in Krebs-Henseleit solution (in mM: 115 NaCl, 25 NaHCO3, 5 KCl, 10 glucose, 1.2 CaCl_2_, and 1.2 MgCl_2_ at pH 7.4) and gassed with a mixture of 95% O_2_-5% CO_2_. Transepithelial voltage (*Vte*) across monolayers was clamped to 0 mV, and a set voltage pulse of 1 mV was applied across cell sheets for 200 ms every 5 s. The *Isc* and transepithelial resistance (*Rte)* across cell monolayers were recorded using Acquire and Analyze Software (Physiological Instruments). After *Isc* and *Rte* stabilized, 10 μM amiloride was added to the apical side and the difference between the post-amiloride *Isc* subtracted from the pre-amiloride *Isc* to determine the amiloride sensitive *Isc*.

### Western Blotting

Western blot analysis was performed as previously described (Flores et al., 2011). Cellular (40 μg) and kidney (20 μg) protein lysates were isolated as described above, resolved electrophoretically, and transferred to Immobilon filters (Millipore, Billerica, MA, United States). Filters were cut horizontally along specific molecular weight bands, blocked in 5–8% non-fat dried milk and 0.05% Tween and immunoblotted with a primary antibody. After being washed, blots were incubated with a horseradish peroxidase-conjugated secondary antibody (Sigma, St. Louis, MO, United States) and bands were visualized by the West Pico enhanced chemiluminescence kit (Pierce, Rockford, IL, United States). Imaging was performed on an iBright FL1500 (Thermo Fisher Scientific, Waltham, MA, United States).

### Reagents

Anti-α-ENaC (1:500; SPC-403), anti-γ-ENaC (1:500; SPC-405) antibodies (StressMarq Biosciences), anti-β-actin (1:1000; A1978) antibody (Sigma Aldrich), anti-pERK (1:10; 9101S for immunostaining) antibody (Cell Signaling), anti-Ki67 (1:1000; VP-MR04; Vectors Labs), and DBA lectin (RS-1032; Vector Labs). U0126 (10 μM) is a MEK1/MEK2 inhibitor which blocks the activation and phosphorylation of ERK.

### Statistics

Data are given as means ± SE (*n* = number of snapwells, or number of animals). Statistical analyses were performed using either unpaired *t*-tests (Excel) or two-way ANOVA (SAS) with appropriate *post hoc* analysis, either Tukey’s or Dunnett’s *post hoc* analysis for cell culture and animal experiments.

## Results

**FIGURE 1:**
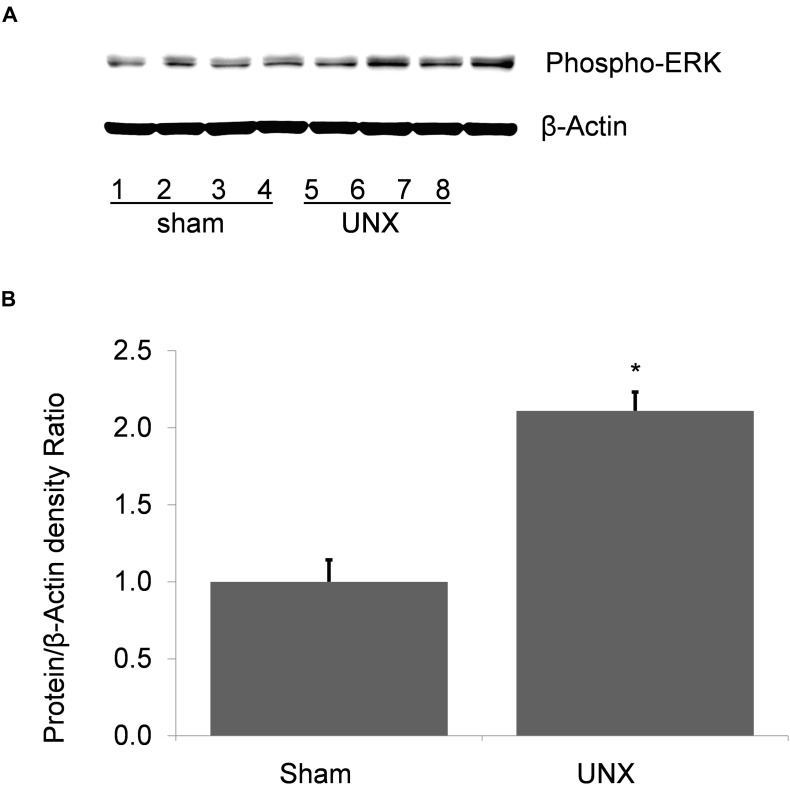
Phospho-ERK abundance in whole kidney protein lysate from sham and unilateral nephrectomy (UNX). **(A)** Western blot of sham (*n* = 4) and unilateral nephrectomy kidney (*n* = 4) shows an increase in steady state abundance of pERK in kidney unilateral nephrectomy. **(B)** Densitometric analysis demonstrates a significant increase of pERK in solitary kidney vs. sham (*, *p* < 0.001).

**FIGURE 2:**
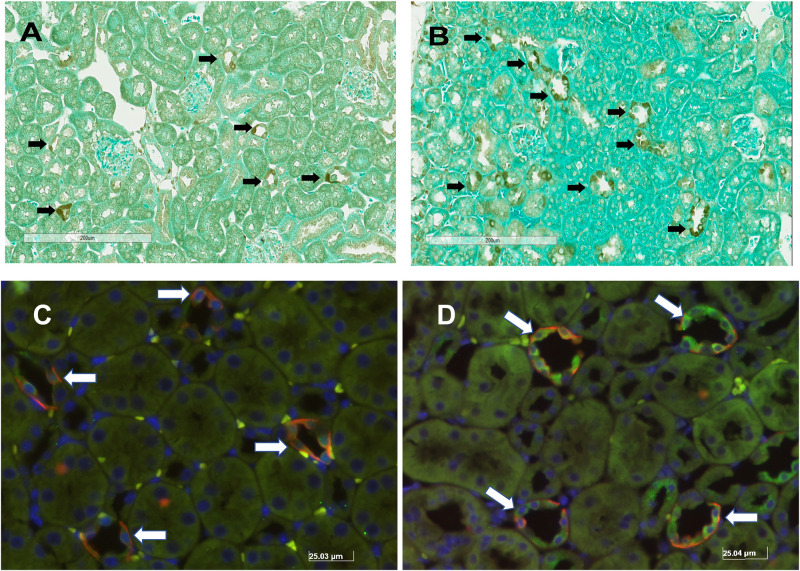
Immunohistochemical pERK staining in murine kidney of sham **(A)** and solitary kidney **(B)** and dual immunofluorescence staining of pERK (GREEN) and DBA (RED) in sham **(C)** and unilateral nephrectomy **(D)**. The black arrows identify the cells staining for pERK in the kidneys of sham **(A)** and after nephrectomy **(B)**. Based on the morphology of pERK expressing cells, most of the staining localizes to the distal nephron in the cortex. To identify the tubular segment staining for pERK, sham **(C)** and nephrectomy **(D)** kidney samples were stained with DBA, to identify principal cells, and anti-pERK antibody. The white arrows identify areas of co-localization of DBA and pERK fluorescent signal.

**FIGURE 3:**
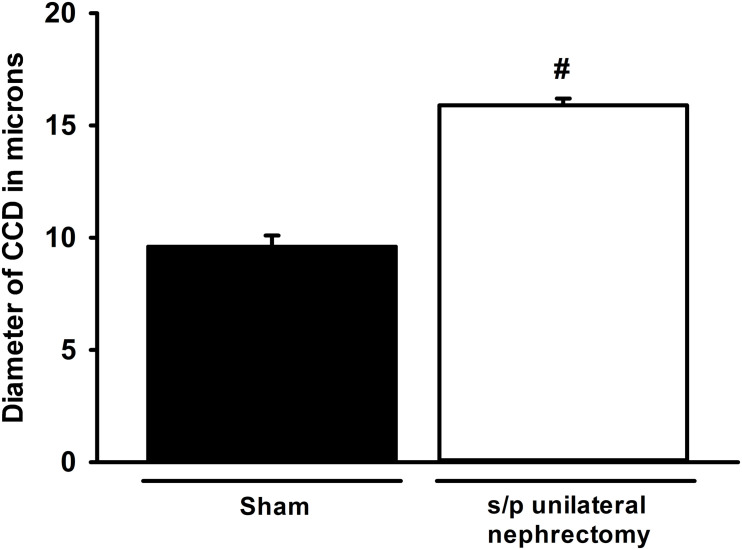
Cortical collecting duct (CCD) diameter is greater in nephrectomized (□) kidneys compared to sham (■) controls. The diameter of CCDs of nephrectomized mice (*n* = 7) increased by approximately 50% compared to sham (*n* = 6) controls (#, *p* < 0.05 compared to sham).

Unilateral nephrectomy is accompanied by compensatory increases in tubular flow rate which leads to augmented FSS and tubular stretch. Prior studies had shown that FSS induces MAPK simulation in collecting duct cells and has been implicated as a regulator of cellular proliferation and ENaC. Western blot of whole kidney protein lysate from sham (*n* = 4) and unilateral nephrectomy after 1 month (*n* = 4; Figure 1A) showed an increase pERK expression in nephrectomized vs. sham mice (Figure 1B). Next, kidneys from sham control and unilateral nephrectomized mice were stained for pERK to identify cells expressing pERK. Though background pERK signal in PTs was noted in sham (Figure 2A) and unilateral nephrectomy (Figure 2B) kidneys, heterogenous expression, where a single cell expressing pERK was adjacent to a cell lacking pERK, appeared to morphologically represent CCD, where two distinct cell populations reside (Figures 2A,B). To confirm the cell type and quantify this difference of pERK expression, double immunofluorescence staining with DBA (red) to identify PCs and anti-pERK (green) antibody was performed on sham (Figure 2C) and nephrectomized (Figure 2D) kidneys. PERK co-localized to CCD cells of the kidney so, quantification was performed on kidneys in six sham and seven nephrectomized mice. The CCD diameter of sham kidneys averaged 9.6 ± 0.5 μ while the CCD’s of nephrectomized mice averaged 15.9 ± 0.3 μ (Figure 3; #, *p* < 0.05).

**FIGURE 4:**
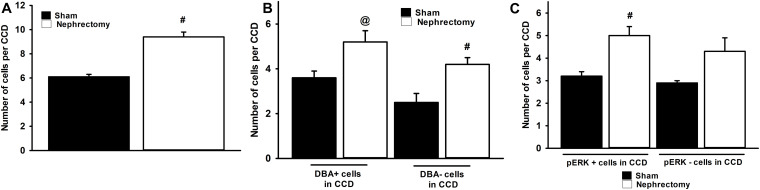
The CCDs of nephrectomy kidney specimens (*n* = 7) contain an absolute increase in number of cells **(A)**, DBA positive and negative cells **(B)**, and pERK expressing cells **(C)** than sham (*n* = 6) controls. The kidneys of mice that received nephrectomy 4 weeks prior to euthanasia contained more cells in the CCD than sham controls **(A)**. A significant increase in DBA negative cells was noted in the nephrectomized mice vs. sham controls (**B**, ANOVA, #, *p* < 0.05) while a trend to increased DBA positive cells was observed in nephrectomized mice (@; *p* = 0.0588). The number of cells that express pERK in the CCD **(C)** was greater nephrectomy specimens than sham (ANOVA, #, *p* < 0.05) but no increase in the number of pERK negative cells in CCD of nephrectomy specimens was noted.

**FIGURE 5:**
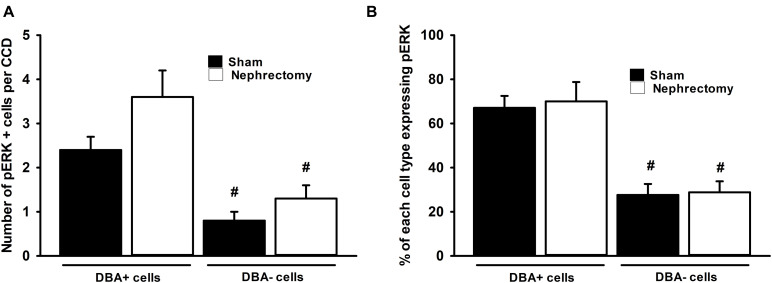
No difference in pERK expression was seen in DBA positive or negative cells between sham (*n* = 6) and nephrectomized (*n* = 7) mice **(A)** and ratio pERK expression in each cell type was unchanged **(B)**. Though overall pERK expression was greater in CCDs of nephrectomized mice, pERK expression in DBA positive or negative cells did not differ in sham compared to nephrectomized mice **(A)**; however, the overall expression of pERK was less in DBA negative compared to positive cells (ANOVA, #; *p* < 0.05 compared to the DBA positive cells in sham or nephrectomy). Also the percent of pERK expression in DBA positive or negative cells did not change based on the sham or nephrectomy status of the CCD **(B)**; however, the percent of pERK expression in DBA negative cell was consistently less than DBA positive cell regardless of sham or nephrectomy status (ANOVA, #; *p* < 0.05 compared to the DBA positive cells in sham or nephrectomy).

The number and type of cells that comprise the CCD in sham and nephrectomized mice was then evaluated. The average number of cells in the CCD increased from 6.1 ± 0.2 cells/CCD in sham kidneys to 9.4 ± 0.4 cells/CCD in nephrectomized mice (Figure 4A; #, *p* < 0.05). DBA positive and negative cells were counted in CCDs of sham and unilateral nephrectomy kidney samples (Figure 4B, ANOVA, *p* < 0.05). The DBA negative cells increased in unilateral nephrectomy compared to sham (Figure 4B, 2.5 ± 0.4 vs. 4.2 ± 0.3 cells/CCD; #, *p* < 0.05) while DBA positive cells also tended to increase in unilateral nephrectomy (Figure 4B, 3.6 ± 0.3 vs. 5.2 ± 0.5 cells/CCD; @, *p* = 0.0588). Next, the number of pERK positive and negative cells was measured per CCD in sham and unilateral nephrectomy (Figure 4C; ANOVA, *p* < 0.05), as an indicator of total pERK activation. The number of pERK expressing cells increased in the nephrectomized mice (3.2 ± 0.2 vs. 5.0 ± 0.4 cells/CCD; Figure 4C, #, *p* < 0.05) compared to sham, while non-pERK expressing cells did not change significantly in nephrectomized kidneys (2.9 ± 0.1 vs. 4.3 ± 0.6 cells/CCD; Figure 4C, *p* = 0.09). While the number of DBA and pERK co-expressing cells did not significantly change between sham and nephrectomy; the DBA negative cells expressed less pERK in either condition, sham or nephrectomy (Figure 5A, #, *p* < 0.05 compared to respective condition). The ratios of pERK expressing DBA positive or negative cells did not change between sham and nephrectomy (Figure 5B).

**FIGURE 6:**
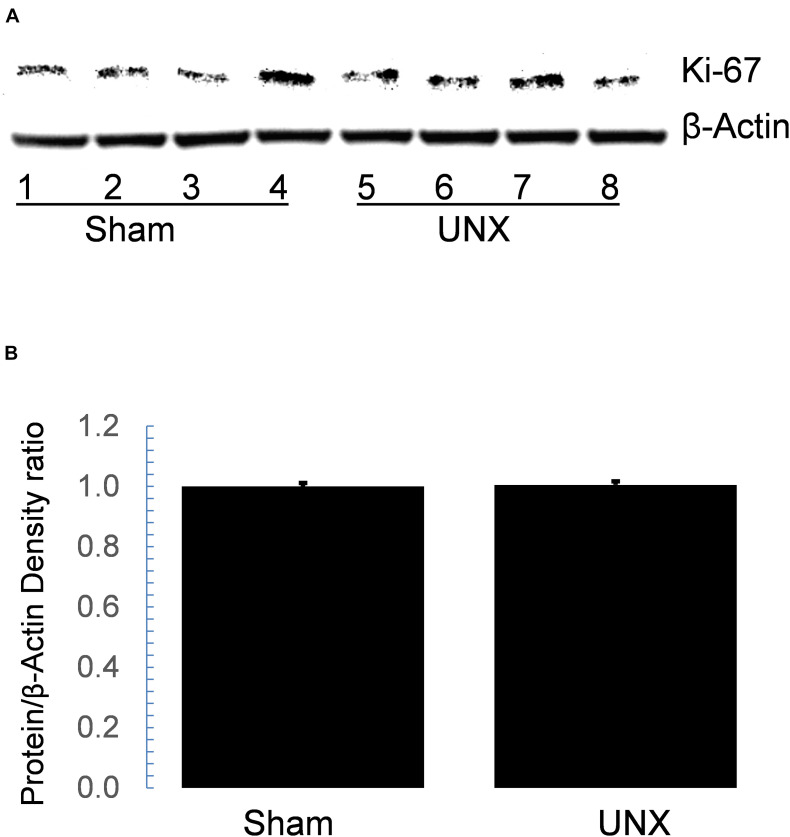
Western blot of Ki67 in whole cell protein lysate did not differ between sham and unilateral nephrectomy (UNX). Steady state abundance of Ki67 protein on immunoblot **(A)** of whole kidney lysate from sham (*n* = 4) and unilateral nephrectomy (*n* = 4) did not differ **(B)**.

Functionally, pERK expression is associated with cellular proliferation, so we tested whether Ki67 expression is increased in whole kidney protein lysate from sham and unilateral nephrectomy. Immunoblotting showed that Ki67 expression did not differ between sham and unilateral nephrectomy at 1-month post-nephrectomy (Figures 6A,B). Immunohistology showed minimal Ki67 staining in either sham or unilateral nephrectomy (data not shown).

**FIGURE 7:**
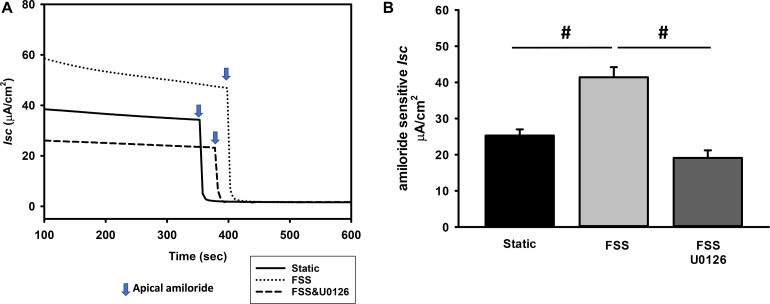
Amiloride sensitive *Isc* is stimulated by an ERK dependent mechanism as demonstrated in a single experiment **(A)** and the composite data **(B)**. MpkCCD cells were exposed either to static, FSS, or FSS with 10 μM U0126 **(A)**. After the *Isc* stabilized, 10 μM amiloride was administered to the apical compartment and the difference in *Isc* before and after amiloride measured. Amiloride *Isc* increased in cells exposed to 0.4 dynes/cm^2^ of FSS (#, *p* < 0.05) compared to static cells and this response was suppressed by U0126 (#, *p* < 0.05) compared to FSS alone.

In addition, the pERK pathway has also been implicated in regulation of ENaC dependent Na transport (Shi et al., 2002; DiPetrillo et al., 2004; Soundararajan et al., 2005, 2007). To test the effects of FSS induced ERK on ENaC dependent Na transport, mpkCCD cells were grown to confluence on snapwell semi-permeable supports and exposed to either static or 0.4 dyne/cm^2^ of FSS for 24 h prior to measuring amiloride sensitive short-circuit current (*Isc*) in an Ussing Chamber. Amiloride sensitive *Isc* increased to 41.4 ± 2.8 μA/cm^2^ (*n* = 22) in FSS exposed cells compared to static cells (25.3 ± 1.7 μA/cm^2^; *n* = 21) and the *Isc* reduced to 19.1 ± 2.1 μA/cm^2^ (*n* = 18) in shear exposed cells treated with U0126, an ERK inhibitor (Figures 7A,B).

**FIGURE 8:**
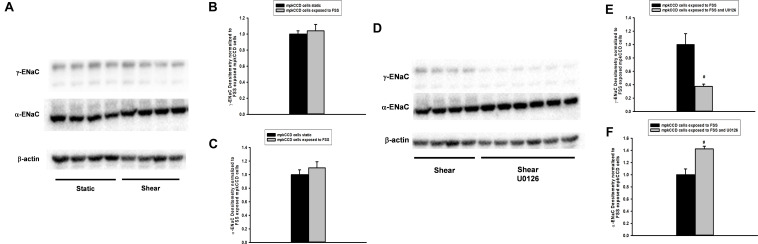
Fluid shear stress (FSS) does not alter abundance of α- or γ-ENaC in whole cell protein lysate **(A–C)**, but treatment with U0126 reduces γ-ENaC and raises α-ENaC expression **(D–F)**. MpkCCD cells grown in plastic plates were either exposed to static (*n* = 8) conditions or FSS of 0.4 dynes/cm^2^ for 24 h (*n* = 9) and then whole cell protein isolated. Immunoblotting **(A)** showed that γ- and α-ENaC subunit **(B,C)** expression was unaffected by FSS. On the other hand, steady state γ-ENaC protein expression was reduced in FSS and U0126 (*n* = 6; #, *p* < 0.05) exposed mpkCCD cells compared shear (*n* = 4) controls **(D,E)**; however, α-ENaC expression increased in the same FSS and U0126 treated cells **(D,F)**.

Next, mpkCCD cells were grown to confluence, exposed to static or FSS conditions, and then whole cell protein lysate immunoblotted. α-ENaC (Figures 8A,C) and γ-ENaC (Figures 8A,B) subunit expression did not differ between static and FSS. To evaluate the effect of ERK inhibition on ENaC protein expression, mpkCCD cells were grown under FSS conditions either exposed to vehicle or 10 μM U0126 for 24 h and immunoblotting performed. γ-ENaC expression in whole protein lysate was reduced in U0126 treated cells vs. controls (Figures 8D,E), while α-ENaC expression increased in FSS exposed cells treated with U0126 (Figures 8D,F).

## Discussion

Biomechanical forces play important roles in renal compensation after nephron loss. Though seminal studies have elucidated how nephron loss increases intraglomerular and oncotic pressure which lead to a rise in single nephron GFR, less is understood about the compensatory affects in tubules. To look at this question, immunohistologic changes were compared between sham and unilateral nephrectomy kidneys and amiloride sensitive *Isc* measured in static and FSS exposed mpkCCD cells. This study identified several novel findings: (1) pERK expression is increased in unilateral kidney, (2) ∼50% increase in diameter of CCDs of solitary kidneys vs. sham control, (3) increase in CCD cell number in unilateral nephrectomy vs. controls, (4) greater pERK expression in CCDs of unilateral nephrectomies, and (5) greater pERK expression in DBA positive than negative cells. The upregulated pERK signaling was not associated with increased Ki67 dependent proliferation in kidneys 1 month after nephrectomy, though an increase in CCD cell number was noted. ENaC dependent *Isc* in mpkCCD cells showed that (1) chronic FSS (for 24 h) induces ERK dependent, amiloride sensitive *Isc* in mpkCCD cells, and (2) ERK inhibition reduces γ-ENaC and raises α-ENaC expression in FSS exposed cells. These observations illustrate pERK signaling is stimulated in CCD of kidney 1-month after nephrectomy and, likely reflects, enhanced FSS induced regulation of cation transport, rather than effects on proliferation.

The hydrodynamic and physiologic effects of nephron loss have been studied extensively in the unilateral nephrectomy rodent model. In rodents, unilateral nephrectomy led to a 12% increase in distal tubule luminal diameter as determined by micropuncture (Hayslett et al., 1968), while in the unilateral nephrectomy mouse model a 50% in CCD diameter was noted. This discrepancy may seem quite large; however, studies performed using two-photon *in vivo* microscopy in rodent kidney showed 45% increase in distal tubule diameter with IV saline injection, implying significant compliance of the distal nephron under high tubular flow (Cortell et al., 1973; Carrisoza-Gaytan et al., 2014).

Though renal tubular hypertrophy is the major response of the contralateral kidney to nephrectomy, cellular proliferation and mitosis also play critical roles, especially in young animals after nephrectomy (Soukupova et al., 1975). DNA synthesis has been noted as early as 6 h post-nephrectomy and cell division noted from 2 days to several days later (Threlfall et al., 1967; Toback and Lowenstein, 1974). At 2 weeks, the solitary kidney has ∼25% higher DNA content than the normal kidney and this increase in DNA content is accompanied by a 25% increase in new cells of the contralateral kidney (Threlfall et al., 1967; Heine and Stocker, 1972; Soukupova et al., 1975). Soon after nephrectomy there are increases in proliferative gene expression including c-fos and c-myc, though which cells express these genes is unknown (Beer et al., 1987; Sawczuk et al., 1990). These seminal studies suggest a proliferative response of the kidney to nephrectomy. However, the proliferative events occur within hours to days after nephrectomy so the increase in CCD cell number likely represents proliferation immediately post-nephrectomy and not active proliferation 1 month later. Therefore, the role of greater pERK abundance at 1 month (Figure 1) does not act as a stimulus for proliferation and is confirmed by minimal Ki67 expression in kidney 1-month post-nephrectomy.

In addition to cellular compensation, functional adaptation occurs which includes increases in GFR and intrarenal alterations in Na transport to maintain Na balance. After unilateral nephrectomy, the GFR in the solitary kidney increases ∼40% and leads to a concurrent rise in the filtered Na load. Micropuncture studies in rodents show no difference in tubular fluid reabsorption in the PT between sham and unilateral nephrectomized rats (Diezi et al., 1976). On the other hand, distal delivery of salt and water nearly doubles requiring a nearly 90% increase in the reabsorptive capacity, implying a change in Na transport distally (Hayslett, 1979). The fractional excretion of Na doubles to due to the fact the single kidney will need to excrete the same quantity of Na as two kidneys; however, the filtered load of Na increases to a much greater extent requiring increased Na reabsorptive capacity.

The mechanism or signal that leads to these adaptive changes is unknown. The robust induction of pERK expression in whole kidney and CCD epithelia, specifically, 1 month after nephrectomy most likely reflects flow mediated pERK regulation of amiloride sensitive Na transport. *In vitro* FSS of 0.4 dynes/cm^2^ for 24 h doubles the amiloride sensitive *Isc* in mpkCCD cells and ERK inhibition prevents this response. These findings are consistent with studies by Muto et al. (1994) who measured the electrical properties of CCD at 3, 6, and 24 h post-nephrectomy and compared their properties to sham controls. CCD transepithelial voltage increased at 3, 6, and 24 h post-nephrectomy compared to sham controls and the amiloride sensitive voltage doubled at 6 and 24 h post-nephrectomy compared to sham controls, implying an increase in ENaC dependent Na transport (Muto et al., 1994).

Though FSS increased amiloride sensitive *Isc*, no change in the molecular expression of the α- or γ-ENaC subunits was observed. However, flow or FSS across ENaC increases the channel’s open probability (Carattino et al., 2004). The Satlin lab demonstrated that acutely raising tubular flow rate from 1 to 5 nL/min.mm in CCDs induces a four-fold increase in Na absorption; primarily due to an increase open channel probability since inhibitors of channel insertion did not affect flow mediated Na absorption (Morimoto et al., 2006). Therefore, an increase in open probability due to FSS may be enough to enhance ENaC dependent Na transport. An increase in the number of apical ENaC channels may also occur, but this was not tested in the mpkCCD model. On the other hand, ERK inhibition reduces γ-ENaC expression to effectively repress apical heterotrimeric ENaC expression and suppress amiloride sensitive *Isc*. Further studies are necessary to elucidate the molecular mechanism underlying FSS regulation of ERK dependent Na transport.

On the other hand, renal cortical γ-ENaC protein expression was reduced in whole protein lysate and biotinylated cell surface proteins of unilateral nephrectomy 24 h after surgery compared to sham controls (Ernandez et al., 2018). In mpkCCD cells exposed to 2 dynes/cm^2^ and, using a Millicell device to measure potential differences and resistance, the amiloride sensitive *Isc* was inferred. FSS at 24 h reduced the normalized amiloride *Isc* by ∼60% with attendant reductions in γ-ENaC protein expression. Why were there differences in FSS induced amiloride sensitive *Isc* compared to this study? It may be related to the level of FSS (0.4 vs. 2 dynes/cm^2^) or to the technique (Millicell measurement of transepithelial voltage and resistance vs. voltage clamp Ussing). Nonetheless, both studies were internally consistent.

Several limitations of our study should be noted. Though we identify increases in pERK expression in CCD and whole kidney lysate of unilateral nephrectomy, its specific *in vivo* physiologic effect on cation transport is only speculative. *In vitro* studies of mpkCCD cells show FSS induces ERK stimulation of ENaC mediated *Isc*, but this has not been confirmed *in vivo* or *ex vivo*. CCD cell number, CCD diameter, and pERK expression are limited by the methods we employed to perform these analyses. To enhance accuracy, a single observer performed all the analyses, and a total of 13 kidneys analyzed. In addition, other investigators have shown that ERK inhibits ENaC dependent Na transport (Shi et al., 2002; Soundararajan et al., 2005, 2007) while we show that FSS enhances ERK dependent Na transport. The differential effects of ERK regulation on ENaC maybe context-specific, for example EGF mediated ERK activation inhibits ENaC, while TNF dependent ERK activation stimulates amiloride dependent Na transport (DiPetrillo et al., 2004).

Nephron loss with concomitant adaptive changes are physiologic responses to maintain GFR, electrolyte balance, and acid-base balance. Though much is known about the initial steps of adaptation, the signals and specific pathways to induce these effects remain elusive. The ERK signaling pathway is activated in CCD of kidneys at last 1 month after nephrectomy and, we speculate, this pathway plays an important role in adaptation. Our *in vitro* studies demonstrate that FSS induces ERK dependent amiloride sensitive Na absorption in a mpkCCD cells which we speculate plays an important role in CCD adaptation after nephrectomy.

## Data Availability Statement

The raw data supporting the conclusions of this article will be made available by the authors, without undue reservation.

## Ethics Statement

The animal study was reviewed and approved by Institutional Animal Care and Use Committee (IACUC), Safety Subcommittee, and R&D Committee as the Veterans Affairs Medical Center (Kansas City, MO, United States).

## Author Contributions

RRe, NM, JM, and KD contributed by performing different aspects of the experiments. TY performed the statistical analyses on the data. MS and TS developed the sham and unilateral nephrectomy mouse model and whole kidney tissue protein derived from these models. RRo designed the experiments, reviewed the data, and wrote the manuscript. All authors contributed to the article and approved the submitted version.

## Conflict of Interest

The authors declare that the research was conducted in the absence of any commercial or financial relationships that could be construed as a potential conflict of interest.

## References

[B1] Beer D. G. Zweifel K. A. Simpson D. P. Pitot H. C. (1987). Specific gene expression during compensatory renal hypertrophy in the rat. *J. Cell Physiol.* 131 29–35. 10.1002/jcp.1041310106 2883191

[B2] Carattino M. D. Sheng S. Kleyman T. R. (2004). Epithelial Na+ channels are activated by laminar shear stress. *J. Biol. Chem.* 279 4120–4126. 10.1074/jbc.m311783200 14625286

[B3] Carrisoza-Gaytan R. Liu Y. Flores D. Else C. Lee H. G. Rhodes G. (2014). Effects of biomechanical forces on signaling in the cortical collecting duct (CCD). *Am. J. Physiol. Renal Physiol.* 307 F195–F204. 24872319 10.1152/ajprenal.00634.2013 PMC4152160

[B4] Cortell S. Gennari F. J. Davidman M. Bossert W. H. Schwartz W. B. (1973). A definition of proximal and distal tubular compliance. Practical and theoretical implications. *J. Clin. Invest.* 52 2330–2339. 10.1172/jci107422 4727462 PMC333038

[B5] Diezi J. Michoud P. Grandchamp A. Giebisch G. (1976). Effects of nephrectomy on renal salt and water transport in the remaining kidney. *Kidney Int.* 10 450–462. 10.1038/ki.1976.132 1011539

[B6] DiPetrillo K. Coutermarsh B. Soucy N. Hwa J. Gesek F. (2004). Tumor necrosis factor induces sodium retention in diabetic rats through sequential effects on distal tubule cells. *Kidney Int.* 65 1676–1683. 10.1111/j.1523-1755.2004.00606.x 15086906

[B7] Du Z. Duan Y. Yan Q. Weinstein A. M. Weinbaum S. Wang T. (2004). Mechanosensory function of microvilli of the kidney proximal tubule. *Proc. Natl. Acad. Sci. U S A.* 101 13068–13073. 10.1073/pnas.0405179101 15319475 PMC516518

[B8] Du Z. Yan Q. Duan Y. Weinbaum S. Weinstein A. M. Wang T. (2006). Axial flow modulates proximal tubule NHE3 and H-ATPase activities by changing microvillus bending moments. *Am. J. Physiol. Renal Physiol.* 290 F289–F296. 16144961 10.1152/ajprenal.00255.2005

[B9] Ernandez T. Udwan K. Chassot A. Martin P. Y. Feraille E. (2018). Uninephrectomy and apical fluid shear stress decrease ENaC abundance in collecting duct principal cells. *Am. J. Physiol. Renal Physiol.* 314 F763–F772. 28877879 10.1152/ajprenal.00200.2017

[B10] Flores D. Battini L. Gusella G. L. Rohatgi R. (2011). Fluid shear stress induces renal epithelial gene expression through polycystin-2-dependent trafficking of extracellular regulated kinase. *Nephron Physiol.* 117 27–36. 10.1159/000321640 PMC2997441 21109758

[B11] Flores D. Liu Y. Liu W. Satlin L. M. Rohatgi R. (2012). Flow-induced prostaglandin E2 release regulates Na and K transport in the collecting duct. *Am. J. Physiol. Renal Physiol.* 303 F632–F638. 22696602 10.1152/ajprenal.00169.2012 PMC3468495

[B12] Gao Y. Stuart D. Takahishi T. Kohan D. E. (2018). Nephron-specific disruption of nitric oxide synthase 3 causes hypertension and impaired salt excretion. *J. Am. Heart Assoc.* 7:e009236. 10.1161/JAHA.118.009236 PMC6064857 29997131

[B13] Hayslett J. P. (1979). Functional adaptation to reduction in renal mass. *Physiol. Rev.* 59 137–164. 10.1152/physrev.1979.59.1.137 220646

[B14] Hayslett J. P. Kashgarian M. Epstein F. H. (1968). Functional correlates of compensatory renal hypertrophy. *J. Clin. Invest.* 47 774–799. 10.1172/jci105772 5641618 PMC297228

[B15] Heine W. D. Stocker E. (1972). Regeneration of kidney parenchyma under normal and pathological conditions. *Beitr. Pathol.* 145 89–99. 5014744

[B16] Hong N. J. Garvin J. L. (2014). Endogenous flow-induced superoxide stimulates Na/H exchange activity via PKC in thick ascending limbs. *Am. J. Physiol. Renal Physiol.* 307 F800–F805. 25080525 10.1152/ajprenal.00260.2014 PMC4187046

[B17] Lenihan C. R. Busque S. Derby G. Blouch K. Myers B. D. Tan J. C. (2015). Longitudinal study of living kidney donor glomerular dynamics after nephrectomy. *J. Clin. Invest.* 125 1311–1318. 10.1172/jci78885 25689253 PMC4362245

[B18] Liu Y. Flores D. Carrisoza-Gaytan R. Rohatgi R. (2015). Cholesterol affects flow-stimulated cyclooxygenase-2 expression and prostanoid secretion in the cortical collecting duct. *Am. J. Physiol. Renal Physiol.* 308 F1229–F1237. 25761882 10.1152/ajprenal.00635.2014 PMC4451326

[B19] Lyon-Roberts B. Strait K. A. van Peursem E. Kittikulsuth W. Pollock J. S. Pollock D. M. (2011). Flow regulation of collecting duct endothelin-1 production. *Am. J. Physiol. Renal Physiol.* 300 F650–F656. 21177779 10.1152/ajprenal.00530.2010 PMC3064134

[B20] Morimoto T. Liu W. Woda C. Carattino M. D. Wei Y. Hughey R. P. (2006). Mechanism underlying flow stimulation of sodium absorption in the mammalian collecting duct. *Am. J. Physiol. Renal Physiol.* 291 F663–F669. 16638910 10.1152/ajprenal.00514.2005

[B21] Muto S. Ebata S. Asano Y. (1994). Short-term effects of uninephrectomy on electrical properties of the cortical collecting duct from rabbit remnant kidneys. *J. Clin. Invest.* 93 286–296. 10.1172/jci116958 8282799 PMC293764

[B22] Pandit M. M. Inscho E. W. Zhang S. Seki T. Rohatgi R. Gusella L. (2015). Flow regulation of endothelin-1 production in the inner medullary collecting duct. *Am. J. Physiol. Renal Physiol.* 308 F541–F552. 25587122 10.1152/ajprenal.00456.2014 PMC4360032

[B23] Repetti R. L. Meth J. Sonubi O. Flores D. Satlin L. M. Rohatgi R. (2019). Cellular cholesterol modifies flow mediated gene expression. *Am. J. Physiol. Renal Physiol.* 317 F815–F824. 31364378 10.1152/ajprenal.00196.2019 PMC6843042

[B24] Rieg T. Bundey R. A. Chen Y. Deschenes G. Junger W. Insel P. A. (2007). Mice lacking P2Y2 receptors have salt-resistant hypertension and facilitated renal Na+ and water reabsorption. *FASEB J.* 21 3717–3726. 10.1096/fj.07-8807com 17575258

[B25] Sawczuk I. S. Olsson C. A. Hoke G. Buttyan R. (1990). Immediate induction of c-fos and c-myc transcripts following unilateral nephrectomy. *Nephron* 55 193–195. 10.1159/000185951 2113996

[B26] Shi H. Asher C. Chigaev A. Yung Y. Reuveny E. Seger R. (2002). Interactions of beta and gamma ENaC with Nedd4 can be facilitated by an ERK-mediated phosphorylation. *J. Biol. Chem.* 277 13539–13547. 10.1074/jbc.m111717200 11805112

[B27] Soukupova M. Hnevkovsky P. Najbrt J. (1975). Effect of age on kidney hyperplasia in the rat after unilateral nephrectomy. *Adv. Exp. Med. Biol.* 53 297–305. 10.1007/978-1-4757-0731-1_24 1119340

[B28] Soundararajan R. Wang J. Melters D. Pearce D. (2007). Differential activities of glucocorticoid-induced leucine zipper protein isoforms. *J. Biol. Chem.* 282 36303–36313. 10.1074/jbc.m707287200 17956870

[B29] Soundararajan R. Zhang T. T. Wang J. Vandewalle A. Pearce D. (2005). A novel role for glucocorticoid-induced leucine zipper protein in epithelial sodium channel-mediated sodium transport. *J. Biol. Chem.* 280 39970–39981. 10.1074/jbc.m508658200 16216878

[B30] Srivastava T. Celsi G. E. Sharma M. Dai H. McCarthy E. T. Ruiz M. (2014). Fluid flow shear stress over podocytes is increased in the solitary kidney. *Nephrol. Dial. Transplant.* 29 65–72. 10.1093/ndt/gft387 24166460 PMC3888310

[B31] Terzi F. Ticozzi C. Burtin M. Motel V. Beaufils H. Laouari D. (1995). Subtotal but not unilateral nephrectomy induces hyperplasia and protooncogene expression. *Am. J. Physiol.* 268 F793–F801. 7539585 10.1152/ajprenal.1995.268.5.F793

[B32] Threlfall G. Taylor D. M. Buck A. T. (1967). Studies of the changes in growth and DNA synthesis in the rat kidney during experimentally induced renal hypertrophy. *Am. J. Pathol.* 50 1–14. 10.1159/000469397 6017684 PMC1965166

[B33] Toback F. G. Lowenstein L. M. (1974). Thymidine metabolism during normal and compensatory renal growth. *Growth* 38 35–44. 4207235

