# Supplementary figures and images for: Unilateral Nephrectomy Stimulates ERK and Is Associated With Enhanced Na Transport
Source: Front Physiol. 2021 Feb 3;12:583453. doi: 10.3389/fphys.2021.583453 (PMC7901926; doi:10.3389/fphys.2021.583453)

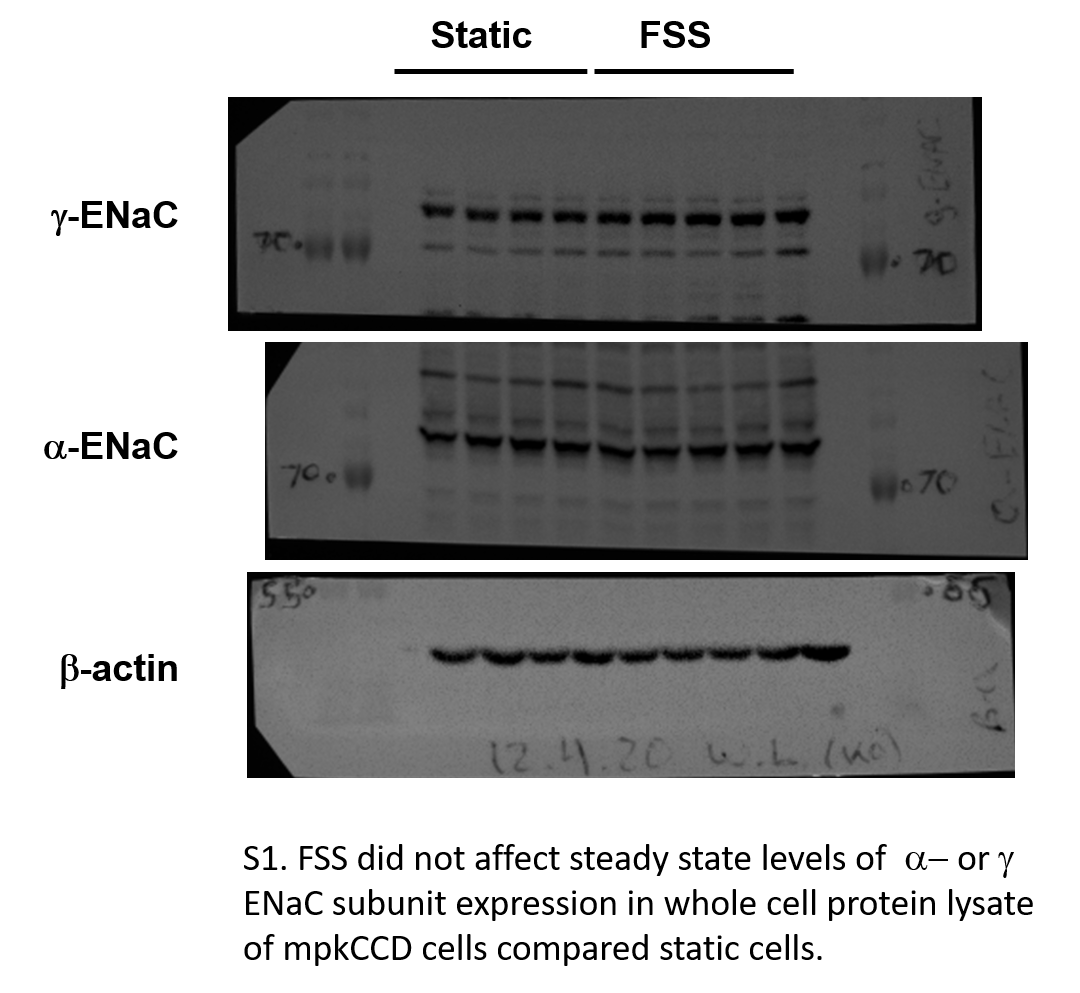

Supplement: Supplementary file 1 [file Image_1.TIF]

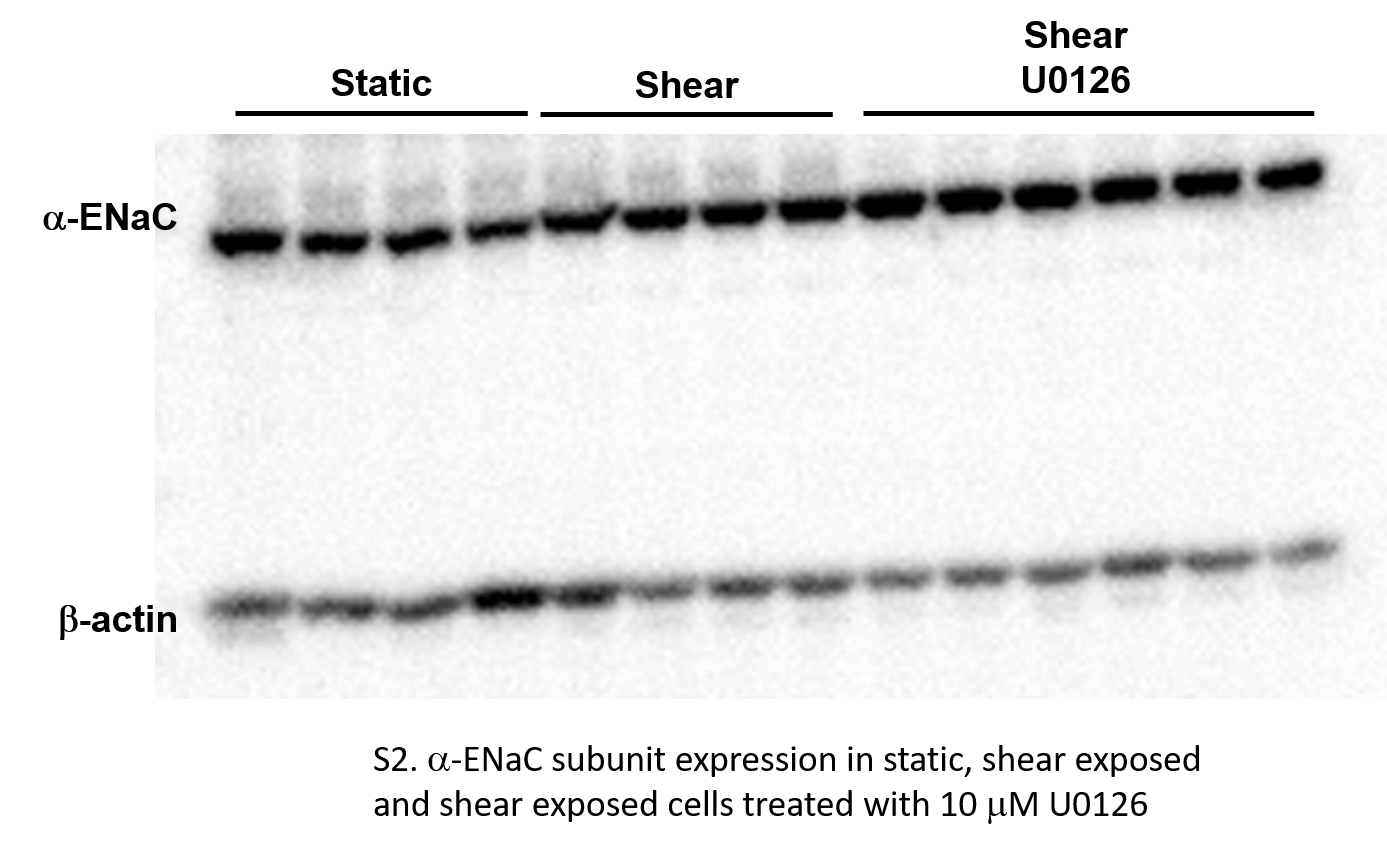

Supplement: Supplementary file 2 [file Image_2.TIF]

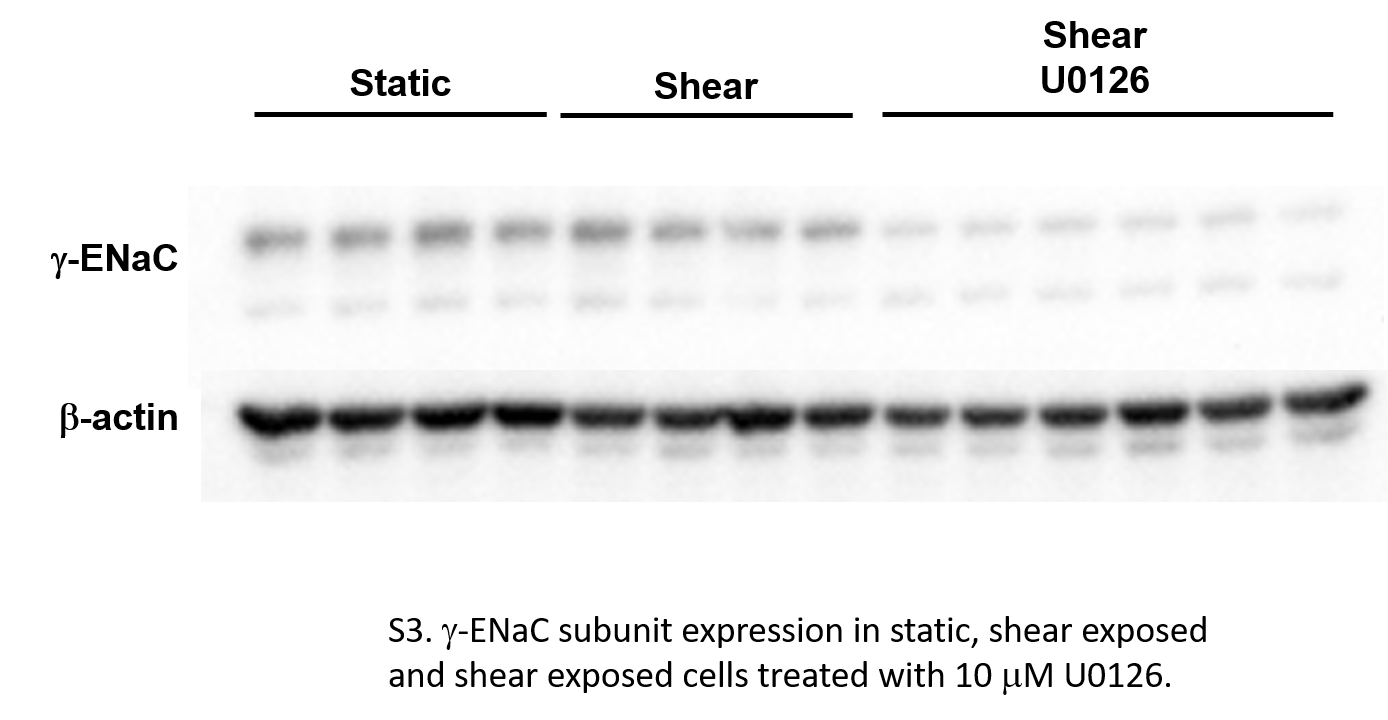

Supplement: Supplementary file 3 [file Image_3.TIF]
